# Latent Profile Analysis of Older Adults by Volunteer Status

**DOI:** 10.1093/geroni/igaf122.3802

**Published:** 2025-12-31

**Authors:** Elnaz Abaei, Shannon Jarrott

**Affiliations:** Iowa State University, Ames, Iowa, United States; The Ohio State University, Columbus, Ohio, United States

## Abstract

Volunteering is generally associated with purposeful activity and social connection, which supports well-being among older adults. Intergenerational volunteering offers additional benefits by supporting principles to reframe aging. Utilizing HRS data, we explored older adult volunteer profiles and outcomes. The sample included 13,135 older adults (M age = 67.26, SD = 10.70), mostly female (58.0%), White/Caucasian (79.0%), and married (59.6%), with a mean12.77 years of education. Using latent profile analysis (LPA), three distinct classes emerged. Class 1 (non-volunteers, 74.6%) showed the least favorable psychological and functional outcomes, with higher loneliness, depressive symptoms, perceived control, and lower mastery and life satisfaction. Class 2 (general volunteers, 20.0%) showed the most favorable profile across indicators. Class 3 (intergenerational volunteers, 5.4%) had a mixed profile, with high mastery and life satisfaction like general volunteers, but elevated loneliness and depressive symptoms like non-volunteers. ANOVA results supported distinctions, showing significant differences across groups in loneliness (F(2, 12,253) = 134.53), perceived control constraints (F(2, 12,498)= 120.39), depression (F(2, 12,686) = 126.93), mastery (F(2, 12,541) = 51.00), life satisfaction (F(2, 12,705) = 106.54), and ADL difficulties (F(2, 13,113) = 89.21), all ps < .001. Small effect sizes (omega-squared = .008–.021) are still meaningful given the large sample, reflecting differences in older adults’ lived experiences. Next, growth mixture models will compare trajectories across profiles to examine change over time. Findings highlight broad benefits of volunteering, particularly general volunteering, including the potential psychological value of intergenerational volunteering as a means to positively reframe aging from youth’s and older volunteers’ perspectives.

